# Comparison of vaginal microbiota sampling techniques: cytobrush versus swab

**DOI:** 10.1038/s41598-017-09844-4

**Published:** 2017-08-29

**Authors:** Anita Mitra, David A. MacIntyre, Vishakha Mahajan, Yun S. Lee, Ann Smith, Julian R. Marchesi, Deirdre Lyons, Phillip R. Bennett, Maria Kyrgiou

**Affiliations:** 1 Institute of Reproductive and Developmental Biology, Surgery and Cancer, Imperial College London, London, W12 0NN UK; 2 Department of Obstetrics & Gynaecology - West London Gynaecological Cancer Centre, Imperial College NHS Trust, London, W2 1NY UK; 3 Department of Biosciences, Cardiff University, Cardiff, CF10 3AX UK; 4 Centre for Digestive and Gut Health, Surgery and Cancer, Imperial College London, London, W2 1NY UK

## Abstract

Evidence suggests the vaginal microbiota (VM) may influence risk of persistent Human Papillomavirus (HPV) infection and cervical carcinogenesis. Established cytology biobanks, typically collected with a cytobrush, constitute a unique resource to study such associations longitudinally. It is plausible that compared to rayon swabs; the most commonly used sampling devices, cytobrushes may disrupt biofilms leading to variation in VM composition. Cervico-vaginal samples were collected with cytobrush and rayon swabs from 30 women with high-grade cervical precancer. Quantitative PCR was used to compare bacterial load and Illumina MiSeq sequencing of the V1-V3 regions of the 16S rRNA gene used to compare VM composition. Cytobrushes collected a higher total bacterial load. Relative abundance of bacterial species was highly comparable between sampling devices (R^2^ = 0.993). However, in women with a *Lactobacillus*-depleted, high-diversity VM, significantly less correlation in relative species abundance was observed between devices when compared to those with a *Lactobacillus* species-dominant VM (p = 0.0049). Cytobrush and swab sampling provide a comparable VM composition. In a small proportion of cases the cytobrush was able to detect underlying high-diversity community structure, not realized with swab sampling. This study highlights the need to consider sampling devices as potential confounders when comparing multiple studies and datasets.

## Introduction

Cervical cancer is a disease that has become largely preventable thanks to screening programmes that allow detection and treatment of pre-invasive disease (cervical intraepithelial neoplasia; CIN)^1^. Oncogenic subtypes of the human papilloma virus (HPV) are the sole causative agent of both CIN and cervical cancer^2^. HPV infection is very common with the lifetime risk of acquiring any HPV infection exceeding 80%^3^, but only in persistent, chronic infection that CIN and cervical cancer may develop over several years to decades^4^. Despite major advances in the understanding of the natural history of HPV infection and cervical disease, we are currently unable to predict the fate of infections and/or pre-invasive lesions.

Analysis of the emerging evidence has lead us to conclude that vaginal microbiota (VM) plays a role in the natural history of HPV infection, and subsequent disease^5–8^. VM composition can be broadly classified into five community state types (CSTs). CST-I, -II, -III and -V are all characterised by one dominant *Lactobacillus* species whereas CST IV is characterised by a high diversity, *Lactobacillus*-deplete community^9^. CST-IV, and to some extent CST-III (*L. iners* dominated), have been associated with increased acquisition and persistence of HPV infection^5^ and increased severity of CIN disease status^7, 8, 10^. The majority of existing data in the literature is derived from cross-sectional cohorts, limiting reported correlations between vaginal microbiota, HPV infection, cervical dysplasia and carcinogenesis to associations that lack causal inference. Longitudinal samples stored in existing cytology biobanks may provide a unique resource that permits temporal assessment and identification of causal associations between the VM, HPV infection and cervical disease.

Rayon swabs are a common device for muscosal sampling and are widely used for next-generation sequencing-based analyses of cervico-vaginal microbial composition^5, 8, 10–12^. However, in the context of CIN and cervical cancer, biobanked samples are typically collected using a cytobrush, which exfoliate the top layer of cervical epithelial cells for detection of dysplasia by cytological analysis using light microscopy and are specifically designed to sample the transformation zone of the cervix; the area where HPV infects and causes dysplastic lesions and invasive cancers^13^. For this reason they may be superior to swabs due to their ability to have a greater surface area contact with the cervical epithelium, ensuring the bacteria in closest contact with this mucosal surface are collected. Furthermore, biofilms of densely adherent bacteria can be present in the vagina, particularly in the case of bacterial vaginosis (BV)^14^. A relatively soft-tipped swab may be unable to disturb these biofilms resulting in sampling of primarily planktonic bacteria not in direct contact with the cervical epithelium. It is also plausible that differences in absorbance and exfoliation between the two sampling devices could lead to variation in the composition of the VM. Previous studies have compared sampling techniques in the nasal sinuses (swab versus biopsy)^15^, and ileum (brush versus biopsy)^16^ and found no significant difference in relative abundance, richness or diversity of bacterial species. A comparison of swabs and cytobrushes used for vaginal microbiota sampling and subsequent analysis by sequencing has not previously been conducted.

## Results

Thirty premenopausal, non-pregnant women with histologically-proven high-grade squamous intraepithelial lesions﻿ (HSIL) were recruited in the colposcopy clinic between July 2014 and April 2015. Patient characteristics are detailed in Supplementary Table 1.

Two samples were taken from each woman during the same vaginal examination by a single clinician (AM), providing a total of 60 samples. There was no difference in mean storage duration from sample collection to DNA extraction between the two sample types (swabs: mean 49 weeks, range 22–62 weeks; cytobrushes: mean 50, range 23–63 weeks, p = 0.7015, paired t-test).

### Cytobrushes collect a greater total bacterial load

**Figure 1:**
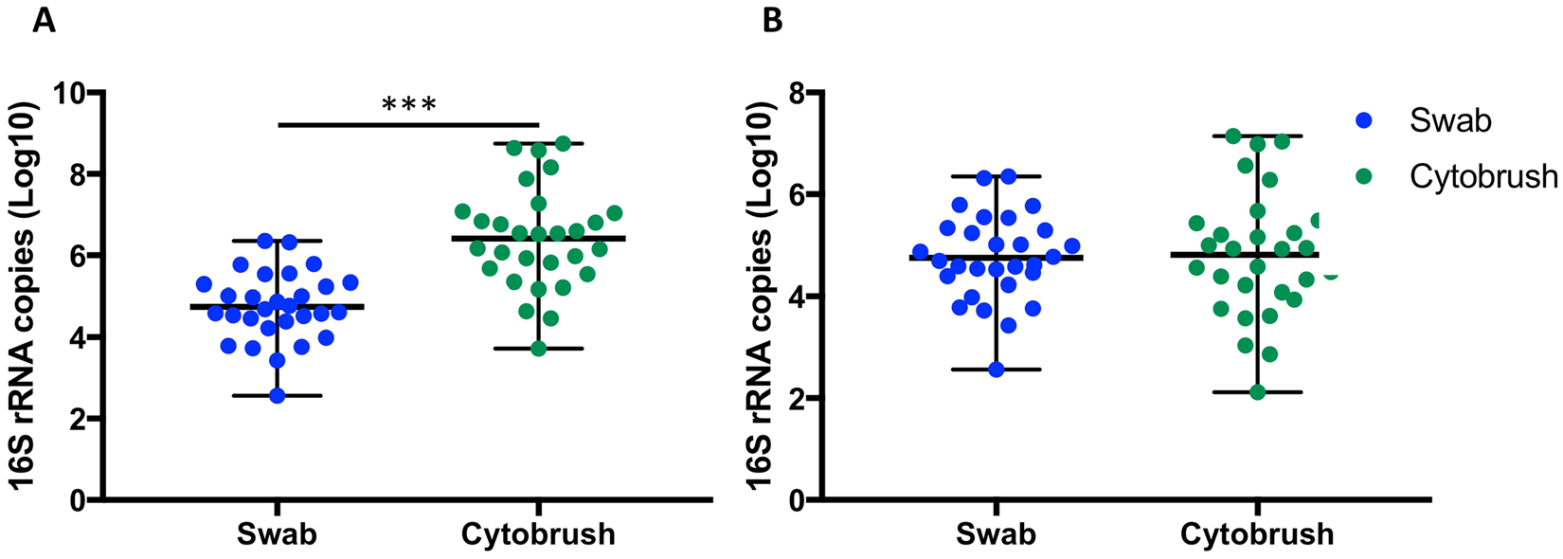
qPCR results. (**A**) Cytobrushes collected a greater total bacterial load compared to swabs (swabs: mean 4.75 log_10_ 16S rRNA gene copies, range 2.56–6.35 log_10_; cytobrushes: mean 6.41 log_10_, range 3.72–8.75 log_10_; p < 0.001) (paired t-test). (**B**) When the bacterial load was normalized to 500 μl with similar amount of medium from the liquid based cytology and Aimes swab solution for Illumina MiSeq sequencing, there was no longer a significant difference between the two techniques (swabs: mean 4.75 log_10_, range 2.56–6.35 log_10_; cytobrushes: mean 4.81 log_10_, range 2.12–7.14 log_10_; p = 0.7361) (Paired t-test).

As estimated using quantitative P﻿CR (qPCR), cytobrushes collected a greater total bacterial load when compared to swabs (swabs: mean 4.75 log_10_ 16S rRNA gene copies, range 2.56–6.35 log_10_; cytobrushes: mean 6.41 log_10_, range 3.72–8.75 log_10_; p < 0.001, paired t-test) (Fig. 1a, Table 1). However, when bacterial load was corrected for volume of storage media, this difference was no longer significant (swabs: mean 4.75 log_10_, range 2.56–6.35 log_10_; cytobrushes: mean 4.81 log_10_, range 2.12–7.14 log_10_; p = 0.7361, paired t-test)(Fig. 1b, Table 1). A total of 500uL was therefore used for further sequencing studies to ensure comparable bacterial DNA loads.

**Table 1:** Results of qPCR and sequencing data analysis.

	Swabs (n = 30)	Cytobrushes (n = 30)	p-value
**Total bacteria load**			
Total bacterial load collected using sampling technique, *Log*_10_ *16*S *rRNA copies (mean, range)*	4.75, 2.56–6.35	6.41, 3.72–8.75	<0.0001
Total bacterial load used for 16S rRNA sequencing, *Log*_10_ *16*S *rRNA copies (mean, range)*	4.75, 2.56–6.35	4.81, 2.12–7.14	0.7361
**Richness and diversity indices**			
Species observed	20, 3.00–72.00	14, 3.00–64 .00	0.8109
Inverse Simpson index	0.77, 0.01–2.39	0.63, 0.01–2.28	0.9125
**Community state types**, *n/N (%)*			
CST I	7/30 (23.3)	7/30 (23.3)	1.000
CST II	3/30 (10.0)	2/30 (6.7)	>0.9999
CST III	14/30 (46.7)	12/30 (40.0)	0.7948
CST IV	5/30 (16.7)	8/30 (26.7)	0.5321
CST V	1/30 (3.3)	1/30 (3.3)	1.000
**Taxa exclusively identified by sampling technique**			
	*- Achromobacter denitrificans*	*- Sphingopyxis chilensis*	*−*
		*- Comamonas* spp. unclassified	
		*- Brevundimonas diminuta*	
		*- Sphingomonas koreensis*	
		*- Burkholderia fungorum*	
		*- Pseudomonas plecoglossicida*	
		*- Ralstonia insidiosa*	
		*- Arthrobacter oryzae*	

CST: community state type; rRNA: ribosomal RNA; spp.: species.

### Swabs and cytobrushes provide comparable 16S rRNA sequencing results

MiSeq-based sequencing of the V1-V3 hypervariable regions of 16S rRNA genes resulted in a total of 696 582 reads, with an average number of 11 610 reads per sample, and a mean and median read length of 543 and 550 bp respectively. Operational taxonomic units (OTUs) were randomly sub-sampled to the lowest read count of 3942 to avoid sequencing bias, which retained 78% of total OTU counts and > 99% coverage for all samples. Following removal of singletons with less than 10 counts, a total of 70 taxa were identified; 61 in both swab and cytobrush samples, eight exclusively in cytobrushes and one exclusively in swabs (Table 1).

**Figure 2:**
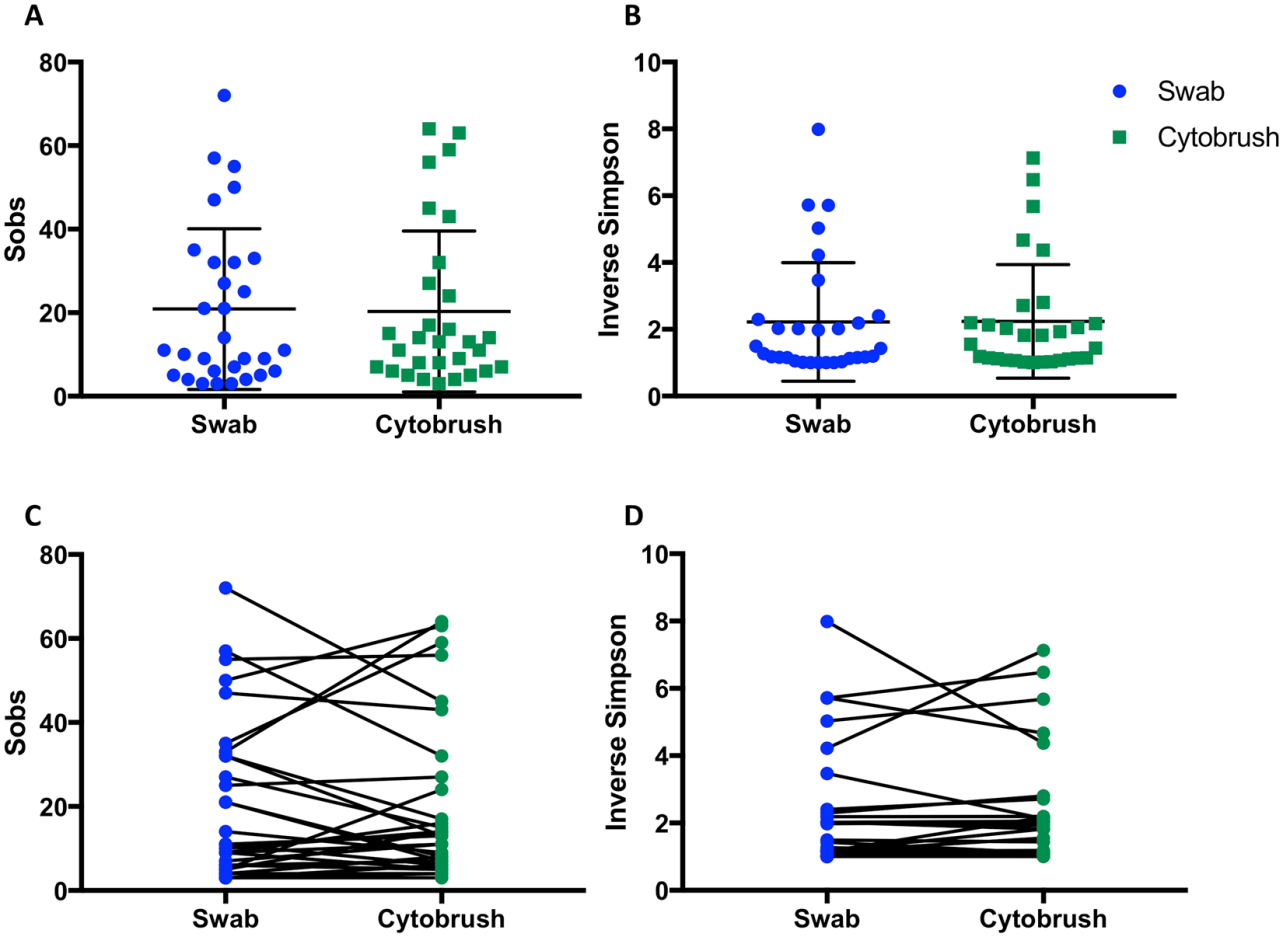
Species richness and diversity indices. Richness, as determined by number of species observed (p = 0.8109) (**A**) and diversity, quantified by inverse Simpson index (p = 0.9125) (**B**) do not differ significantly between the two sampling techniques (paired t-test). Richness (**C**) and diversity (**D**) were similar between swabs and cytobrushes in most women, however where different, the values were not consistently higher or lower with either technique. *Sobs* =S*pecies observed*.

There was no significant difference in richness, as determined by number of species observed (p = 0.8109 paired t-test), or diversity, quantified by inverse Simpson index (p = 0.9125, paired t-test) (Fig. 2A and B, Table 1) between the two sampling techniques, however they were not consistently higher or lower where different (Fig. 2C and D).

**Figure 3:**
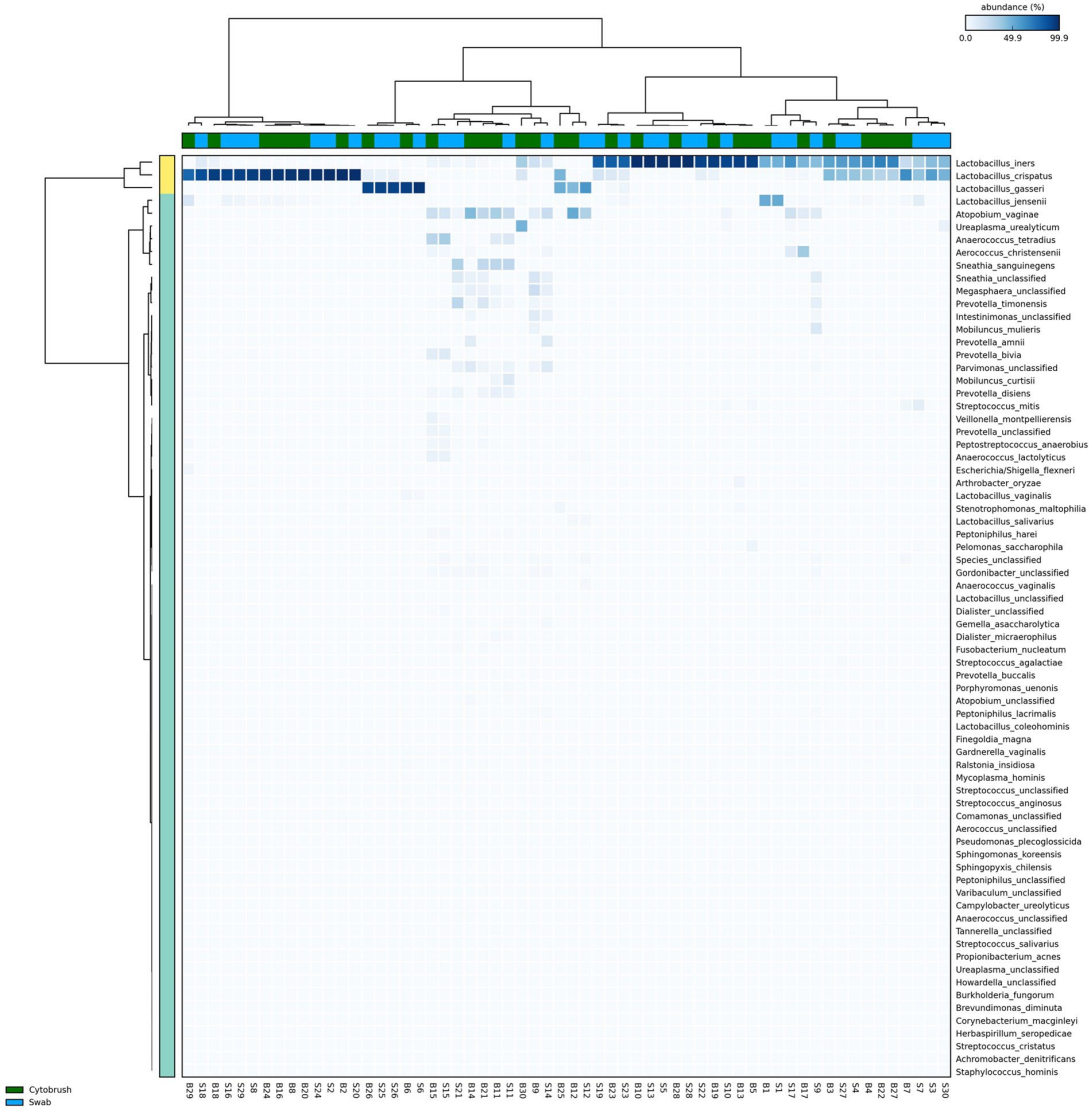
Heatmap. Hierarchical clustering analysis using ward clustering was used to classify samples into community state types (CSTs). There was a 90% concordance in CST classification (27/30 patients) between swab and brush sampling. Three patients with a *Lactobacillus*-spp. dominant vaginal microbiota on swab sampling were subsequently found to have a high-diversity CST IV on cytobrush sampling.

Ward clustering of relative abundance at species level was performed and demonstrated the presence of four of the five previously described CST’s^9^ within the dataset, with CST-V not observed (Fig. 3). Concordance in CST between the two sampling techniques was found in 27 of 30 patients (90%), with discordance in three of 30 (10%). Of these, two patients displayed a *Lactobacillus iners*-dominant (CST-III) structure on the swab and high-diversity *Lactobacillus*-spp. deplete CST-IV on the cytobrush-collected sample. The remaining discordant sample set also showed CST-IV using the cytobrush, but the *Lactobacillus gasseri*-dominant CST-II on the swab. When comparing the entire dataset, this discrepancy between CST’s in the swab and cytobrush-collected samples was not statistically significant (Table 1).

**Figure 4:**
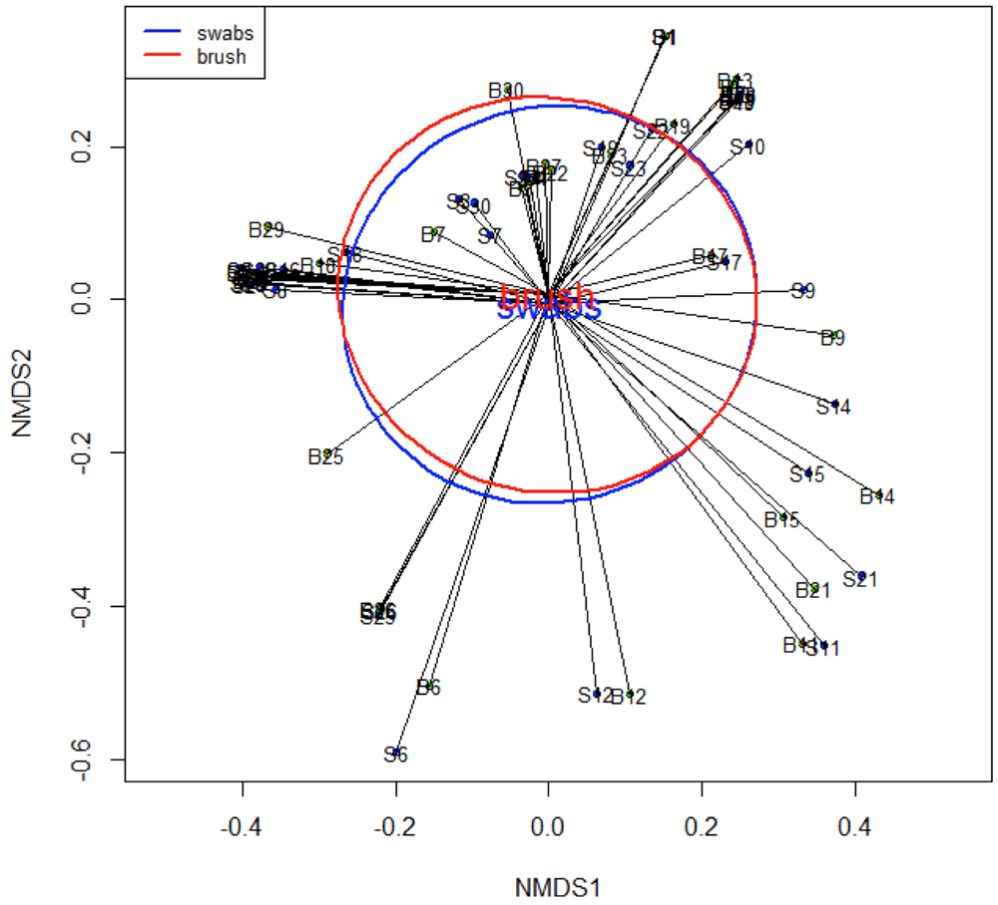
Non-metric multidimensional scaling (NMDS) analysis for paired brush and swab samples. NMDS analysis of the Bray-Curtis dissimilarity matrix revealed no significant difference in community composition between cytobrush- and swab-collected samples. Ellipses represent standard error.

Bray-Curtis index of dissimilarity was used to compare the microbial community structure of the samples collected via the two different techniques (Fig. 4). Visualisation of the dissimilarity matrix using NMDS revealed no difference in the overall community structure between sampling devices (p = 0.99, PERMANOVA test).

**Figure 5:**
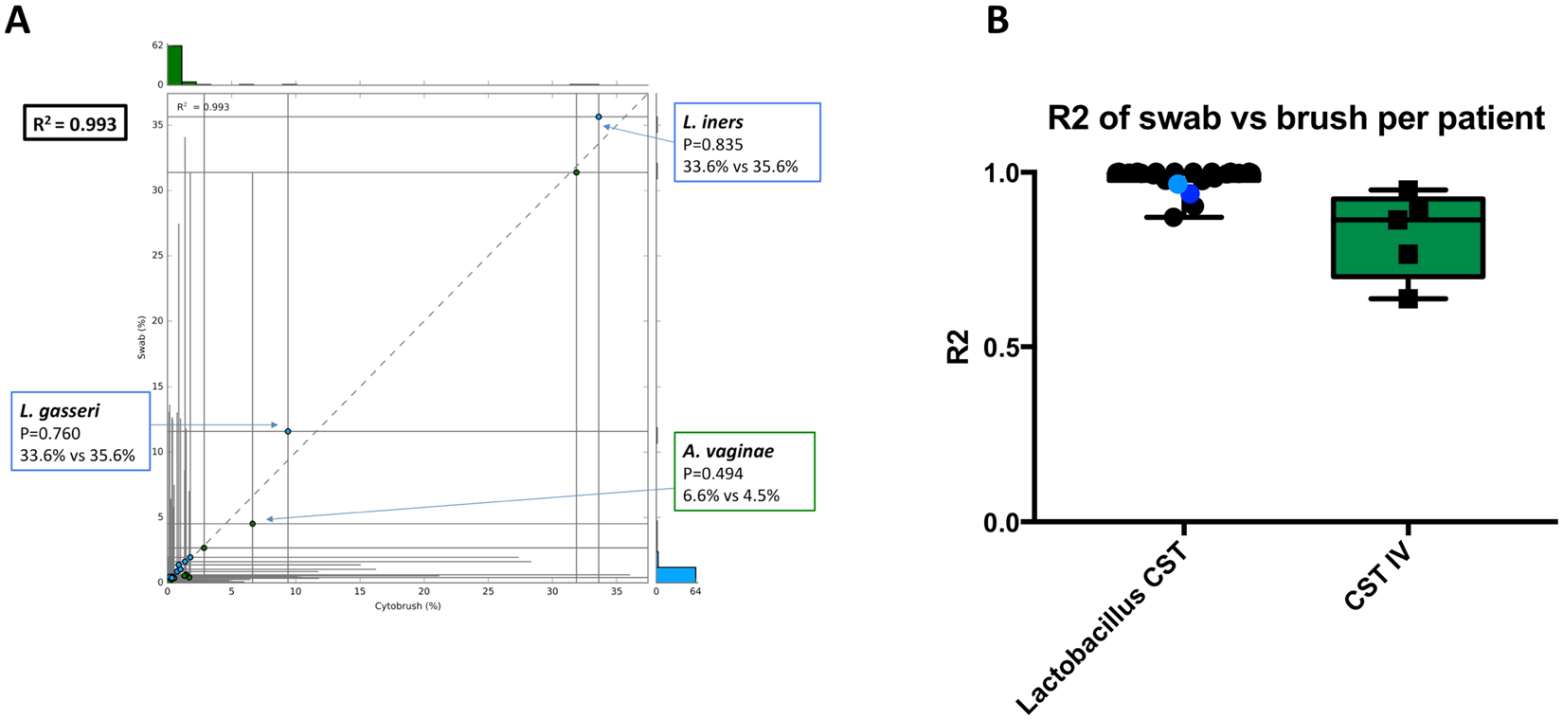
Correlation between sample composition at species level. (**A**) Using a 2-group comparison the correlation between composition at species level was found to be 0.993 (Welch’s t-test). (**B**) Using 2-sample comparison, the correlation between swab and cytobrush samples was significantly less in women with CST IV, compared to those with a *Lactobacillus*-spp. dominant vaginal microbiota (p = 0.0049, Mann-Whitney U test).

A two-group comparison of the different sampling techniques was also performed showing that relative abundance of bacterial taxa was highly comparable between all swabs and cytobrushes (R^2^ = 0.998–0.999 from phylum to genus level, R^2^ = 0.993 at species level) (Fig. 5A). Similarly, high correlation was observed when a paired two-sample comparison was performed to examine individual patient correlation of swab and cytobrush-collected samples (R^2^ = 0.908; range 0.408–1.00). When comparing intra-patient variability between the two sampling techniques, significantly less correlation of species abundance was observed between the two samples in women with CST-IV compared to women with *Lactobacillus* species-dominant VM (*Lactobacillus*-dominant CST mean R^2^ = 0.982 vs. CST-IV mean R^2^ = 0.706, p = 0.0049, Mann-Whitney U test) (Fig. 5). The mean R^2^ values for the individual *Lactobacillus*-dominant CST’s were 0.995 (CST-I), 1.00 (CST-II) and 0.971 (CST-III).

**Figure 6:**
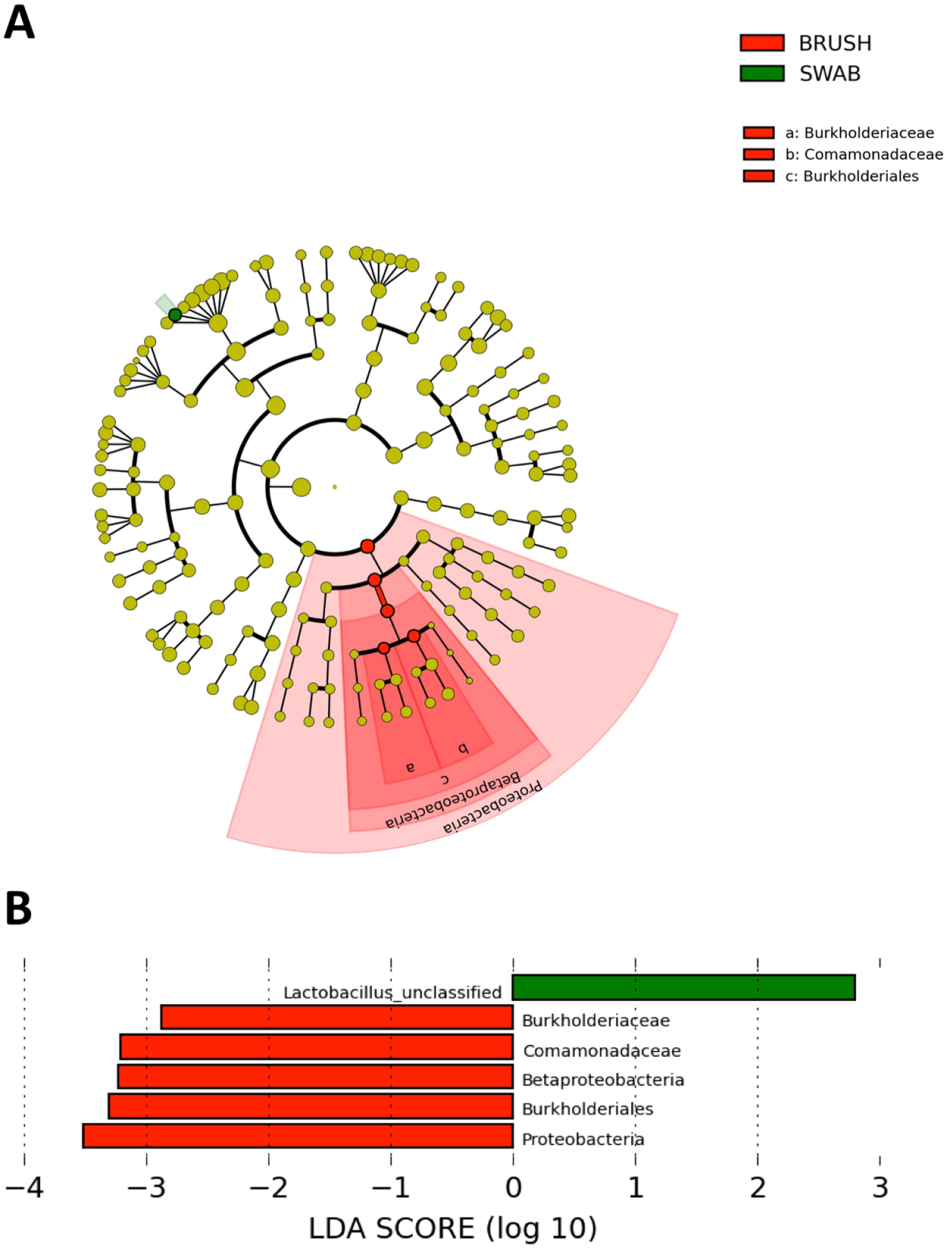
Identification of differentially abundant taxa between swabs and cytobrushes. (**A**) Cladogram representing taxa with different relative abundance according to sampling technique. Size of circle is proportionate to relative abundance of taxon. (**B**) Histogram of the LDA scores computed for features differentially abundant between swab and cytobrush-collected samples (Welch’s t-test). *LDA score: Linear discriminant analysis score*.

LEfSe analysis identified five taxa belonging to the same clade to be significantly over-represented in the cytobrush samples (*Proteobacteria, Betaproteobacteria, Burkholderiales, Burksholderiaceae* and *Comamondaceae*; Fig. 6), although the relative abundance of these taxa was low overall. Taxa attributed to unclassified *Lactobacillus* spp. was over-represented in swabs. Further LEfSe analysis performed on the subgroup of patients with at least one CST-IV sample (n = 8) failed to identify any differentially abundant features.

*Gardnerella vaginalis* qPCR was used to determine whether the choice of 16S rRNA sequencing primers may have influenced the comparison between the two techniques. When comparing 500ul swab carrier fluid to 500 ul LBC fluid, the volume with which comparable bacterial counts are seen (Fig. 1) there is no significant difference in levels of *G. vaginalis*. When a difference between swabs and brushes was observed it was neither consistently higher nor lower (Supplementary Figure 1).

## Discussion

Cross-sectional studies exploring the associations between the VM, HPV infection and cervical pre-invasive and invasive disease have shown that a high-diversity VM, and to a lesser extent *L. iners*-dominant VM’s correlate with increasing cervical disease severity^7, 8, 10^, and in acquisition and persistence of its causative agent HPV^5^. Longitudinal studies are required to infer causality with regards to the role of the human microbiota in oncogenesis^17^. However, the change from a normal healthy cervix, through HPV acquisition, chronic infection resulting in dysplasia and onward neoplastic transformation to invasive cancer takes at least a decade^18^. Biobanks, largely collected as part of cervical screening programmes, contain liquid-based cytology samples collected using cytobrushes, which provide a unique resource of serial samples required to further explore the associations between VM and cervical carcinogenesis. Several techniques have been described in the literature for obtaining samples for the purpose of sequencing bacterial DNA to study the human vaginal microbiota in a variety of patient cohorts, the most common being swabs^19^, but the use of cytobrushes^20^, as well as cervicovaginal lavage^21^, epithelial scrapes and biopsies^22^ has also been reported. Heterogeneity of the vaginal microbiota at different locations throughout the vagina has been documented^23^, however a direct comparison of swabs and cytobrushes taken from the ectocervix has never been described.

Cytobrushes, unlike swabs, are made of polyethylene and lack any absorptive capability. Whilst the suitability of different swab types has not been reported in studies exploring the vaginal microbiota, experiments *in vitro* have shown that a significant difference exists in both absorbance and release of compounds and proteins from different swab types^24^. Cytobrushes have a greater exfoliative ability compared to swabs in the studies on VM, possibly giving them the additional ability to disturb biofilms. We therefore hypothesised that cytobrushes would be associated with higher diversity due to these different properties.

In this study we compared the results obtained using swabs and cytobrushes, from a population of 30 women with HSIL to determine whether these two techniques provide a comparable overview of the structure of the vaginal microbiota. We chose women with high-grade pre-invasive disease as opposed to low-grade or normal controls or a mixture, since our previous study^8^, which included women with various disease severity and healthy controls, indicated that women with HSIL should have good representation of major vaginal CSTs. This allowed us to compare the similarity of the two sample techniques in both low and relatively high-diversity vaginal communities in this pilot study.

Cytobrush sampling collected higher bacterial loads, as assessed using qPCR. Whilst the cervical microbiota has been demonstrated to be similar in composition to the vagina, it may have comparatively lower bacterial load^25^, reinforcing the importance of collecting the greatest possible load. A small aliquot of the total 20 ml LBC solution was used, and we were able to use qPCR to determine the volume to be used to give similar total bacterial load (Fig. 1b, Table 1) to prevent biasing further sequencing experiments with a discrepancy in bacterial load between the two techniques. We have also demonstrated the higher biomass collected by cytobrushes in a separate cohort of 20 further women in whom the mean weight of sample collected by swabs was 50 milligrams (mg), compared to 1560 mg by cytobrushes (unpublished data), which supports that cytobrushes collect a much higher biomass than swabs. The advantage of using only one fortieth of the original sample for 16S rRNA gene analysis leaves the investigator with a large volume remaining with which to do further tests such as cervical cytology, HPV genotyping, and general microbiology reducing the need for extra sampling of women recruited to research studies. However, with the increasing interest in metagenomic/whole genome shotgun sequencing, the cytobrush-collected samples may contain a high load of host DNA, which can be problematic, however this was not assessed in the current study.

Overall our study demonstrated that swab and cytobrush samples provide comparable VM results at all taxonomic levels, as demonstrated by two-group/sample correlation coefficients, hierarchical clustering analysis and Bray-Curtis dissimilarity index. No significant difference in richness or diversity between the two sampling techniques were identified disproving our hypothesis that cytobrush-collected samples would be associated with higher diversity. In spite of this, a greater number of unique taxa were observed in cytobrush samples and LefSe analysis identified *Proteobacteria, Betaproteobacteria, Burkholderiales, Burksholderiaceae* and *Comamondaceae* to be over-represented in the cytobrush-collected samples however, levels of each were present at extremely low abundance. Their presence has not previously been associated with the presence of HPV and cervical disease in studies using swabs for sampling.

Although overall correlation between swab and cytobrush data at an individual level was high, reduced correlation was observed in women with high-diversity CST-IV. LEfSe analysis of this smaller patient subset did not detect any differentially abundant taxa, but this may be due to a lack of statistical power. There was a discrepancy between the CST classification of sequencing data in 3/30 (10%) women, all of whom had a swab sample which clustered with a *Lactobacillus* spp.-dominant CST, but with CST-IV on their cytobrush sample. It is plausible that sampling with a cytobrush disrupts biofilms that are otherwise left intact when sampled with a rayon swab resulting in the isolation of taxa present in planktonic phase. Clearly further studies are required to confirm this, however our data indicates that swab-sampling techniques may be less suitable when studying diseases correlated with highly diverse communities. Cross-sectional data in high-grade pre-invasive cervical disease document high prevalence of dysbiosis^8^ and therefore cytobrush-sampling techniques may reduce sample collection bias in these patients.

Although swabs are considered by some patients to be less invasive than a cytobrush, they are not used to collect samples for cytological screening due to their inability to exfoliate an adequate number of endocervical cells for cytological analysis^26^. Cytobrushes can not only be used for cervical cytology and HPV DNA testing, but we show that they provide a reliable and robust sampling tool for analysis of the vaginal microbiota. It should be noted however, that cytobrushes do not harbour absorbance qualities and thus dual sampling with a swab may provide useful material for analysis the proteomic and metabolic component of cervicovaginal mucosa.

One of the limitations of sequencing is that the results may be influenced by the choice of primer sets^27^. We have used primers for V1-V3 hypervariable regions of 16S rRNA genes, and acknowledge that these may not detect members of the *Bifidobacteriales* order, which includes *Gardnerella vaginalis* ^27^, a species frequently detected in the human vagina^28^. In order to determine whether primer choice influenced our results we performed *G.vaginalis* qPCR, and showed that where this species was detected, there was no significant difference between the two sampling techniques (Supplementary Figure 1), and we therefore do not consider this to be a significant limitation to our conclusions. Furthermore, the collection of both samples was performed during the same vaginal examination in order to ensure identical conditions and allow a direct comparison between the sampling techniques. The cytobrush, which has a greater exfoliative capacity compared to a swab was intentionally collected second to ensure that this does not disturb the biofilms prior to the swab collection. Given the wide surface of the cervix and the amount of discharge found in women, it is unlikely that the gentle tip of the swab would be sufficient to disturb the microbiota in the cytobrush, hence the reason for such a study design.

This report is the first study to compare the VM in women sampled using swabs or cervical cytobrushes. A single clinician collected all samples, in an attempt to minimise the likelihood of intra-study variability in the sampling collection techniques. Our results indicate that resulting sequencing data derived from both sampling devices are comparable, yet cytobrushes permit the collection of a greater bacterial load. This may be in part due to their larger surface area, but these samples can also be used for additional cervical cytology and HPV DNA testing purposes. Looking beyond the current study, the results have implications in possible future attempts to synthesise the existing evidence and integrate existing multiple studies and datasets for the purpose of meta-analysis^29^, as differential technique, device and site of sampling, whilst producing small variability may have a profound confounding effect on larger analyses. Further larger studies are required to confirm the findings of this study.

In conclusion, analysis of our data shows that rayon swabs and polyethylene cervical cytobrushes produce comparable results when comparing the vaginal microbiota composition at species level, and did not show any significant difference in diversity or richness. However, cytobrushes were able to uncover CST-IV VM’s, not demonstrated by the corresponding swab sample in 10% of our sampled population, which may be due to the cytobrush having a greater exfoliative capacity, which could enable biofilm disruption. We have also shown that cytobrushes collect a higher bacterial load, which may reduce the impact of potential sample contamination. These results should be taken into consideration when designing future prospective studies where a high-diversity microbiota may be implicated in disease pathogenesis, and those performing meta-analysis of metagenomic data should consider variation in sampling techniques as a potential confounder. Based on our findings we conclude that cervical cytobrushes are a valid sampling device for collection of samples for 16S rRNA gene analysis, which opens up the possibility of using historical biobank samples for the study of longitudinally collected patient samples.

## Methods

### Study population – Inclusion and Exclusion criteria

Ethical approval was obtained from the National Research Ethics Service Committee London – Fulham (Approval number 13/LO/0126). All experiments were performed in accordance with the approved guidelines and regulations. All patients gave informed consent. We included pre-menopausal non-pregnant women, 18–45 years of age who attended the colposcopy and gynaecology clinics at Imperial College NHS Healthcare Trust with a histologically-proven diagnosis of high-grade squamous intra-epithelial lesion (HSIL). We chose these as opposed to normal controls as we have previously demonstrated that major vaginal CSTs are represented in this patient cohort^8^. Women who were HIV or hepatitis B/C positive, with autoimmune disorders, which received antibiotics or pessaries within 14 days of sampling, or had a previous history of cervical treatment were excluded. Detailed medical and gynaecological history was collected. Ethnicity was self-reported as Caucasian, Asian or Black.

### Sample collection and processing

A swab followed by cytobrush sample was collected from the same patient at the same time-point. During sterile speculum examination without lubricant a swab was first taken from the ectocervix using a BBL^TM^ CultureSwab^TM^ containing liquid Amies with a rayon tip (Becton Dickinson, Oxford, UK) and stored immediately at −80 °C followed by a cytobrush used in the standard manner to collect a cervical sample using the ThinPrep Preservcyt system (Hologic, Crawley, UK) and stored at 4 °C. Whole genomic bacterial DNA was extracted from 500 μl of either the Preservcyt solution (cytobrush) or liquid amies (swab) using a QIAmp *cador* Pathogen Mini kit (Qiagen, Venlo, Netherlands) according to manufacturer’s instructions.

### Quantitative polymerase chain reaction (qPCR)

Quantitative real-time PCR was carried out for quantification of 16S rRNA gene copy number in order to determine the volume of Preservcyt required for sequencing and to compare the bacterial load collected by each technique in total and of *G. vaginalis*. Real-time qPCR was performed with universal BactQUANT 16S rRNA gene primers (Forward primer: 5′-CCTACGGGAGGCAGCA, Reverse primer: 5′-GGACTACCGGGTATCTAATC) (Sigma) with the FAM labeled BactQUANT probe ((6FAM) 5′-CAGCAGCCGCGGTA-3′ (MGBNFQ))^30^ and *G. vaginalis* primers (Forward primer: 5′-GGAAACGGGTGGTAATGCTGG, Reverse primer: 5′-CGAAGCCTAGGTGGGCCATT)^31^ using a SYBR green-based assay on the Applied Biosciences StepOne machine (Thermo Fisher Scientific, Ashford, UK) with StepOne software version 2.3 (Life Technologies). Samples were run in duplicate.

### Illumina MiSeq sequencing of 16S rRNA gene amplicons

The V1–V3 hypervariable regions of 16S rRNA genes were amplified by PCR using a forward and reverse fusion primer as previously described^32^. Sequencing was conducted at Research and Testing Laboratory (Lubbock, TX, USA).

### 16S rRNA gene sequence analysis

Sequence data was analysed in Mothur using the MiSeq SOP Pipeline^33^. Sequence reads were quality checked and normalised to the lowest number of reads. Singleton operational taxonomic units (OTUs) and OTUs < 10 reads in any sample were collated into OTU_singletons and OTU_rare phylotypes respectively, to maintain normalisation and to minimise artefacts. OTUs were defined using a cut off value of 97% and result data analysed using Vegan package within the R statistical package for assessment of microbial composition and diversity (R Development Core Team 2008). OTU taxonomies (from Phylum to Genus) were determined using the ribosomal database project (RDP) MultiClassifier script to generate the RDP taxonomy^34^ while species level taxonomies of the OTUs were determined using the USEARCH algorithm combined with the cultured representatives from the RDP database^35^. Alpha and beta indices were calculated from these datasets with Mothur and R using the Vegan package.

### Statistical analysis

Analysis of statistical differences between the vaginal microbiota of cytobrush- versus swab-retrieved samples collected during the same examination from the same women was performed using the Statistical Analysis of Metagenomic Profiles (STAMP) package^36^. Data were subjected to multivariate analysis using principal component analysis (PCA) and hierarchical clustering analysis (HCA) by nearest neighbour linkage with a clustering density threshold of 0.75. Linear discriminant analysis (LDA) effect size (LEfSe) analysis was used to identify taxa significantly overrepresented in either sampling device, through all taxonomic levels^37^. This analysis was performed using taxonomic relative abundance, with per-sample normalization and default settings for alpha values (0.05) for the factorial Kruskal-Wallis test among classes and pairwise Wilcoxon test between subclasses. A logarithmic LDA score greater than 2 was used to determine discriminative features.

Multivariate dissimilarity analysis was performed using the Vegan package in R. A Bray-Curtis dissimilarity index was constructed using the *vegdist* function. Non-metric multidimensional scaling (NMDS) was further performed using species assignments, and a PERMANOVA was used to perform multivariate ANOVA based on dissimilarities using the *adonis* function.

Fisher’s exact test, Mann–Whitney U tests and t-tests were performed where appropriate using GraphPad Prism v.6.04 (GraphPad Software Inc., California, USA). A p-value less than 0.05 was considered statistically significant.

Public access to sequence data and accompanying metadata can be obtained from the European Nucleotide Archive’s (ENA) Sequence Read Archive (SRA) repository; https://www.ncbi.nlm.nih.gov/sra (accession number PRJEB19346).

## Electronic supplementary material

Supplementary Table 1 & Supplementary Figure 1
