# Supplementary material for: Comparison of vaginal microbiota sampling techniques: cytobrush versus swab
Source: Sci Rep. 2017 Aug 29;7:9802. doi: 10.1038/s41598-017-09844-4 (PMC5575119; doi:10.1038/s41598-017-09844-4)
Supplement: Supplementary file 1 — Supplementary Table 1 & Supplementary Figure 1 [file 41598_2017_9844_MOESM1_ESM.pdf]

Supplementary information

Comparison of vaginal microbiota sampling techniques: cytobrush versus swab

Anita Mitra, David A MacIntyre, Vishakha Mahajan, Yun S Lee, Ann Smith, Julian R. Marchesi, Deirdre Lyons, Phillip R Bennett, Maria Kyrgiou.

Supplementary Table 1. Patient characteristics

**Characteristics**	**n = 30**
**Age, years** Mean (SD, range)	30.8 (4.9, 26 – 43)
**Ethnicity , n/N (%)**	
Caucasian	27/30 (90.0)
Asian	3/30 (10.0)
Black	0/30 (0)
**Parity, n/N (%)**	
Nulliparous	20/30 (66.7)
Parous	10/30 (33.3)
**Smoking status, n/N (%)**	
Current smoker	5/30 (16.7)
Non-smoker	25/30 (83.3)
**Phase of menstrual cycle, n/N (%)**	
Luteal	8/30 (26.7)
Follicular	15/30 (50.0)
Unknown	7/30 (23.3)
**Contraception, n/N (%)**	
Nil	13/30 (43.3)
Condoms	5/30 (16.7)
COCP	5/30 (16.7)
POP	2/30 (6.7)
Copper IUD	1/30 (3.2)
Mirena IUS	2/30 (6.7)
Contraceptive implant	2/30 (6.7)
**Time since last intercourse, n/N (%)**	
>48 hours	26/30 (86.7)
<48 hours	4/30 (13.3)
**Histological severity of lesion**	
CIN2	20/30 (66.7)
CIN3	10/30 (33.3)

CIN: Cervical intraepithelial neoplasia; COCP: Combined oral contraceptive pill; IUD: Intrauterine device; IUS: Intrauterine system; POP: Progesterone-only pill

A B

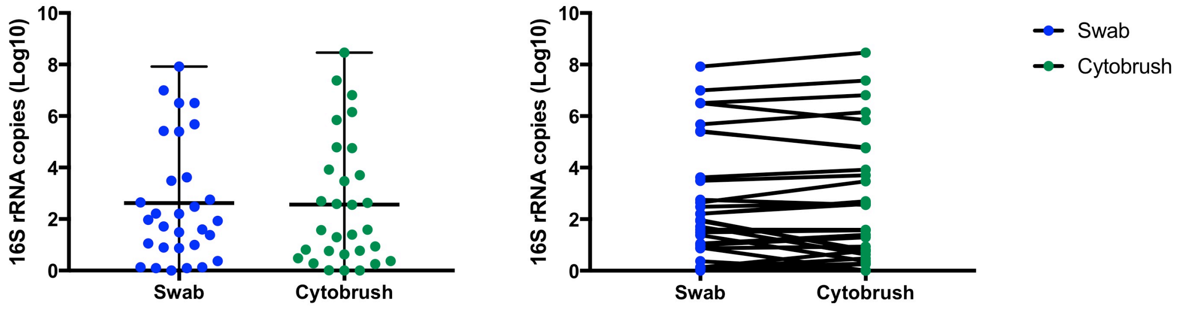

Supplementary Figure 1. Comparison of Gardnerella vaginalis gene copies using qPCR

There was no significant difference between the number of G.vaginalis gene copes present in paired swabs and cytobrush collected samples, normalised for total bacterial load (Figure 1) (p=0.6313) (Paired t-test) (A). Where different the values were not consistently higher or lower with either technique (B).
